# Early Introduction of Multi-Allergen Mixture for Prevention of Food Allergy: Pilot Study

**DOI:** 10.3390/nu14040737

**Published:** 2022-02-09

**Authors:** Antonia Zoe Quake, Taryn Audrey Liu, Rachel D’Souza, Katherine G. Jackson, Margaret Woch, Afua Tetteh, Vanitha Sampath, Kari C. Nadeau, Sayantani Sindher, R. Sharon Chinthrajah, Shu Cao

**Affiliations:** Department of Medicine, Division of Pulmonary, Allergy and Critical Care Medicine, Sean N. Parker Center for Allergy and Asthma Research at Stanford University, Stanford, CA 94304, USA; zoeq@stanford.edu (A.Z.Q.); tliu2236@gmail.com (T.A.L.); rdsouza@zis.ch (R.D.); kjawesomeness3@gmail.com (K.G.J.); mwoch@stanford.edu (M.W.); okobea@gmail.com (A.T.); vsampath@stanford.edu (V.S.); tina.sindher@stanford.edu (S.S.); schinths@stanford.edu (R.S.C.); shucao@stanford.edu (S.C.)

**Keywords:** multi-allergen, early introduction, food allergy, safety, efficacy, prevention

## Abstract

The incidence and prevalence of food allergy (FA) is increasing. While several studies have established the safety and efficacy of early introduction of single allergens in infants for the prevention of FA, the exact dose, frequency, and number of allergens that can be safely introduced to infants, particularly in those at high or low risk of atopy, are still unclear. This 1-year pilot study evaluated the safety of the early introduction of single foods (milk, egg, or peanut) vs. two foods (milk/egg, egg/peanut, milk/peanut) vs. multiple foods (milk/egg/peanut/cashew/almond/shrimp/walnut/wheat/salmon/hazelnut at low, medium, or high doses) vs. no early introduction in 180 infants between 4–6 months of age. At the end of the study, they were evaluated for plasma biomarkers associated with food reactivity via standardized blood tests. Two to four years after the start of the study, participants were evaluated by standardized food challenges. The serving sizes for the single, double, and low dose mixtures were 300 mg total protein per day. The serving sizes for the medium and high dose mixtures were 900 mg and 3000 mg total protein, respectively. Equal parts of each protein were used for double or mixture foods. All infants were breastfed until at least six months of age. The results demonstrate that infants at either high or low risk for atopy were able to tolerate the early introduction of multiple allergenic foods with no increases in any safety issues, including eczema, FA, or food protein induced enterocolitis. The mixtures of foods at either low, medium, or high doses demonstrated trends for improvement in food challenge reactivity and plasma biomarkers compared to single and double food introductions. The results of this study suggest that the early introduction of foods, particularly simultaneous mixtures of many allergenic foods, may be safe and efficacious for preventing FA and can occur safely. These results need to be confirmed by larger randomized controlled studies.

## 1. Introduction

The incidence and prevalence of food allergy (FA) has increased significantly in recent years [1,2,3]. Today, an estimated 7.6% of U.S. children and nearly 11% of adults have reported FAs to at least one food [4,5]. Reactions can be mild, moderate, severe, or life-threatening, with clinical symptoms varying considerably between individuals and over one’s lifetime [6]. Although a number of foods are known to be allergenic, cow’s milk, hen’s egg, peanuts, soy, wheat, tree nuts, fish, and shellfish account for 90% of all FAs [7]. Accidental ingestion rates for peanut, egg, and milk range from 14 to 33% [8], 19–50%, and 17–36%, respectively [9].

There is an urgent and unmet need to prevent and treat FAs. Despite limited data, older guidelines recommended delaying exposure to cow’s milk until 12 months, hen’s egg until 24 months, and peanut, tree nut, and fish until three years [10] to prevent FA. In 2015, results of a large randomized controlled study, the Learning Early About Peanut (LEAP) study, challenged these early guidelines and concluded that early introduction of peanuts significantly reduced the likelihood of developing peanut allergy and, conversely, delayed introduction significantly increased the likelihood of developing peanut allergy in infants. The study also indicated that delayed introduction may lead to impaired immune responses [11]. The benefits of early introduction of allergenic foods for prevention of FA are now supported by a number of randomized controlled trials, particularly for peanut, egg and milk. A meta-analysis found strong evidence of positive benefits of early introduction of allergenic foods [12].

Allergen diversity in infants has also been found to play a role in preventing FA. In 2014, the PASTURE study found that increased diversity of complementary allergenic foods introduced in the first year of life was inversely associated with doctor-diagnosed FA up to 6 years of age, food sensitization, and increased expression of a T regulatory cell marker [13]. Diet diversity was also evaluated in a large prospective study using standardized questionnaires. The study showed that the introduction of each additional allergenic food at 6 and 12 months of age reduced the odds of developing FA over the first 10 years of life by 10.8% and 33.2%, respectively [14]. Systemic reviews suggest that allergen diversity in infancy may be associated with reduced allergy outcomes (including FA) [15,16].

Among children with FA, up to 40% are allergic to multiple foods [5]. This suggests that there is a need to include multiple allergenic foods into the diets of these infants for prevention of FA, as research shows that it is allergen-specific [17]. At the current time, only a few studies have looked at the introduction of multiple allergenic foods for the prevention of FA. In the Enquiring About Tolerance (EAT) study, infants were randomized at three months and sequentially introduced to six allergenic foods (including cow’s milk, peanut, hard-boiled egg, sesame, cod and wheat) vs. to a standard group where infants avoided allergenic foods. Per protocol analysis indicated that the early introduction group had a 67% relative risk reduction of any FA compared to the standard group [18]. While the EAT study showed that it was safe and effective to introduce multiple allergens, the amounts of allergens used were impractical as per the USDA guidelines for daily caloric intake in infants (528, 619, and 806 calories daily for infants of four, six, and 12 months of age, respectively) [19]. Infants in the EAT study were asked to consume the equivalent of 4 g of each allergenic protein per week, which amounted to two small 40- to 60-g portions of cow’s milk yogurt, three rounded teaspoons of peanut butter, 1 small hard-boiled egg, three rounded teaspoons of sesame paste, 25 g of whitefish, and two wheat-based cereal biscuits. There were 10 participants whose families reported food protein induced enterocolitis syndrome (FPIES)-like reactions, seven in the early-introduction group and three in the standard-introduction group. The difference between the two groups was not statistically significant (*p* = 0.34). Holl et al. conducted a blinded randomized placebo-controlled study to determine whether early introduction of multiple allergens simultaneously is acceptable to caregivers and tolerable to any healthy infant. In this study, the 16 most common allergenic foods (peanut, soy, almond, cashew, hazelnut, pecan, pistachio, walnut, wheat, oat, milk, egg, cod, shrimp, salmon, and sesame) were premixed and fed to infants between the ages of 5–11 months, for a total of 480 mg of proteins per day for 28 days. This study had a high completion rate indicating acceptability, and showed no significant difference in safety between the active vs. the placebo group (*p* = 0.76). There were no increases in adverse events and there were no increases in reported IgE-type allergic reactions in the active vs. the placebo group [20]. In fact, the placebo group had allergic reactions likely due to the delayed introduction of allergenic foods.

As evidence increasingly suggested that allergen diversity and early intervention may decrease FA [11,21], infant feeding guidelines were revised, and currently recommend full introduction of diverse allergenic foods at 4 to 6 months [22,23,24]. However, the early and consistent introduction of not just single allergens, but multiple allergens can be difficult to administer in infants. Further research on the number of allergens, amount of allergenic proteins, and length of time allergens need to be consumed is needed to optimize tolerability while providing a convenient and practical method for the early introduction of proteins to infants. Therefore, in the current pilot descriptive study, we conducted a one-year-feeding randomized clinical trial to evaluate the safety of simultaneously introducing a multi-allergen mix vs. double mix vs. single foods vs. no early introduction in infants with high and low risk of allergy. In addition to clinical evaluations of ingestion tolerance via oral food challenges, biomarkers were also measured (allergen-specific IgE and IgG_4_).

## 2. Materials and Methods

Study Design: The pilot study was conducted at the Sean N. Parker Center for Allergy and Asthma Research at Stanford University under an approved Stanford IRB study protocol (8629). This was a descriptive study that was designed to be a pilot study for the preliminary assessment of the safety of a 10-allergen protein mixture. Parents or legal guardians consented for their infants to participate in the study for one year and were given instructions for how to give the daily servings of the food proteins (in powder or flour form).

Flours and powder preparation: The flours and powders for each food were prepared in a Good Manufacturing Practice facility at the Sean N Parker Center for Allergy and Asthma Research at Stanford University. Each flour and powder were obtained by manufacturers that had to meet specifications of low yeast, no salmonella and no *E. coli* content. Each flour and powder was examined for protein integrity by standard SDS protein gel electrophoresis and by protein concentration methods using the standard BioRad assay.

Samples from each lot of allergen and placebo substance received were analyzed upon receipt to confirm that the identity of the material and to ensure that microbial levels fall within acceptable limits prior to use in the manufacture of the drug product. A summary of baseline analyses and their methodology can be found in Table 1. Upon meeting all acceptance criteria, the lot was accepted for use in the study. To prepare the 10-food protein mixture, flours and/or powders were weighed in the GMP facility using standard operating procedures and per mg protein amount, added in a 1:1:1:1:1:1:1:1:1:1 ratio. SDS PAGE gels were also run on the mixtures to ensure all proteins were present at equal ratios.

### 2.1. Substance Identity & Protein Composition

Excluding placebo (oat powder), the active ingredients within each allergen-specific food powder are the combined proteins or singular proteins. For each allergen, the presence and intensity of a readily observable allergenic protein/subunit specific to each allergen substance was confirmed via sodium dodecyl sulfate polyacrylamide gel electrophoresis (SDS-PAGE) after Coomassie Blue staining (Table 2).

Manufacturers were as follows for each powder or flour. Milk: Now REAL foods, Illinois; almond: Honeyville Farms, CA, USA; egg: Honeywell Farms, CA, USA; cashew: nuts.com, NJ; hazelnut and walnut: Holmquist Orchards, CA, USA; oat (placebo) and wheat: Arrowhead Mills, NY, USA; shrimp and salmon: Invico, WA, USA. Peanut: Golden Peanut Company, Alpharetta, GA, USA.

Enrollment and Eligibility: Both high risk infants and low risk infants were enrolled and stratified 1:1. High risk was defined as one first degree relative with FA/atopic dermatitis or two first degree relatives with atopic disease [25]. Exclusion criteria included infants with chronic diseases or known genetic diseases or with known FA. The study was conducted with IRB approval with enrollment between June 2017 and July 2019 and infants between two months and 12 months of age were enrolled (NCT04828603). Participants were randomized (but not blinded) equally: singles (egg, milk, or peanut; *n* = 15 each), doubles in equal parts by mg protein (peanut plus milk, peanut plus egg, or milk plus egg, *n* = 15 each), or a 10-allergen mixture (almonds, cashew, egg, hazelnut, milk, peanut, salmon, shrimp, walnut, wheat) and an age- and sex-matched control group (*n* = 45) which avoided all potentially allergenic foods for the first year of the study. The serving sizes for the 10-allergen mixture were low, medium, or high (i.e., 300 mg per day, *n* = 15; 900 mg per day, *n* = 15; 3000 mg per day, *n* = 15, in equal 1:1 parts of the 10 proteins, respectively). Participants were fed the first serving in the clinic and observed for 2 h post-ingestion. Upon leaving, parents were instructed to observe the participants at-home for the next 4 h and report any adverse events within 24-h post-ingestion. Participants were in the active phase of the study for one year with regular daily servings of their food in their assigned group. The parent or guardian of each participant was asked to fill out a food diary over a seven day period at the end of the one year study to assess the child’s ability to eat table foods from the same foods as in the 10-protein mixture. If a child ate that food as a table food in the week following the end of the one year study period, they was defined as being able to eat that table food. At baseline and at one year, participants underwent a blood draw. Allergen-specific IgE and IgG4 were measured using a standard ImmunoCAP assay (Phadia, Uppsala, Sweden). Oral food challenges (up to 8 g of total protein from the 10-food allergen mixture) were conducted between 2–4 years after the start of the study in a facility with trained personnel with staged, monitored standard methods. Questionnaires were provided at baseline and one year and at the time of the food challenge. 

### 2.2. Statistical Analysis

The study was a pilot descriptive study to test the preliminary safety of a mixture of 10 food allergen proteins in otherwise healthy infants with no current food allergies. The study was not designed to be a phase 2 or phase 3 study, and was not powered to detect a difference in safety between subgroups. Efficacy was measured as an exploratory endpoint and was tested through a standard food challenge and reported here. In addition, bioindicators such as plasma markers were evaluated as exploratory measures and reported here.

The changes of allergen-specific IgE, IgG4, and IgG4 to IgE ratio from baseline to 1 year are depicted as box plots. The Wilcoxon signed rank test was performed to determine whether the change was significant from baseline to 1 year. The Benjamini-Hochberg (BH) procedure was used to control the false discovery rate (FDR) for multiple comparisons among all multi-allergen groups for each marker. Q value was used to denote the adjusted *p* value. The food challenge outcome at one year was illustrated using a bar chart, and the success rate of participants passing the food challenge between groups were compared using a chi-squared test. *p* values were adjusted using BH procedures and denoted as Q values. All tests were two-sided and conducted at the 0·05 level of significance. All analyses were conducted using R software v4.0.3. Descriptive statistics for questionnaire results and demographic information were documented in tables.

## 3. Results

**Figure 1:**
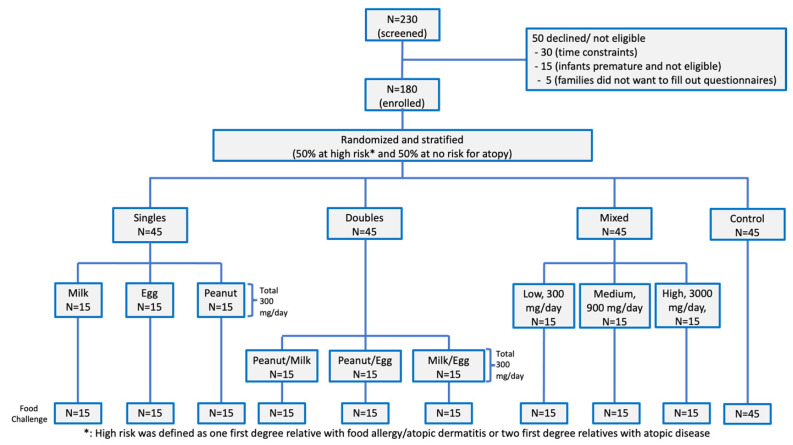
Consort diagram. 180 participants were randomized into three active and one control group. The active phase of the study was for one year and there were no dropouts. Single foods (milk, egg, or peanut); two foods (milk/egg, egg/peanut, milk/peanut), Mixed (milk/egg/peanut/cashew/almond/shrimp/walnut/wheat/salmon/hazelnut at low, medium, or high doses).

One hundred and eighty healthy infants aged 2 months to 12 months were recruited at a single site. Participants were randomized and stratified so that 50% of participants were at high risk for FA, and 50% of participants were not at risk of FA. Each of the active groups were further randomized into specific allergen groups as depicted in the consort diagram (Figure 1). No participant dropped out early from the study. Infants consumed daily proteins for an average of 12.1 ± 3.2 months. There was a 95% adherence rate for each infant. The control group avoided the same 10 food proteins for the first 12 months. All children were breastfed to at least six months of age. All participants had available values (or there were no missing values found) for all tested allergen-specific IgE, IgG4, and food challenge outcomes. 

Patient demographics are shown in Table 3. In this cohort, 149 (83%) reported having atopic dermatitis. Fifty-one percent were of high risk, and this was evenly spread throughout all the cohorts. Fifty-one percent% were female. The median age at the start of the study was six months. Eleven percent of participants were Hispanic, 20% were Asian, 51% Caucasian, 14% African American, and 4% Pacific Islander.

For safety parameters, across all active groups, there were no increases in allergic reactions reported in participants regardless of risk stratification and eczema comorbid condition. Reactions, including all mild skin rashes, were reported in both the control group, *n* = 4 (9%), and active groups, *n* = 11 (8%). None of those with the mild skin rashes had evidence of eczema or documented family history. Eczema (mild, moderate, and severe) decreased in both controls and in all active groups (Table 4) over time. There were no other adverse events such as vomiting, diarrhea, anaphylaxis, wheezing, cough, or epinephrine use within 2 h of consumption of the allergenic foods. The percentage of participants consuming real foods at the beginning and end of the study is shown in Table 5.

**Figure 2:**
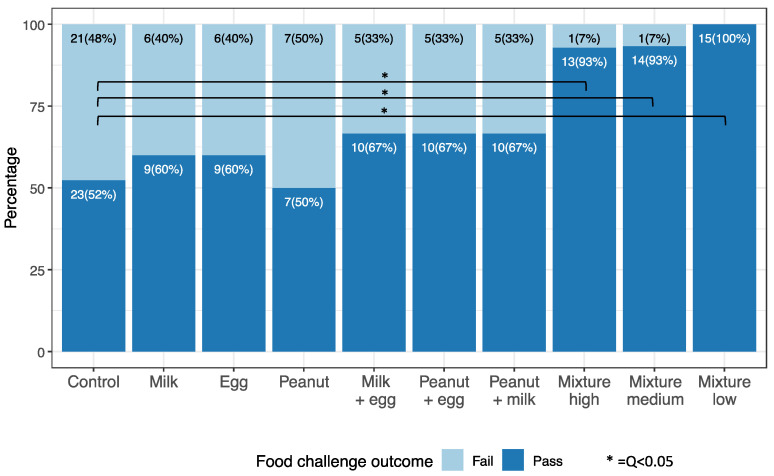
Oral Food Challenges: Food challenge outcome in active (singles, doubles, and mixtures) and control groups 2–4 years after start of study. Oral food challenges (up to 8 g of total protein from the 10-food allergen mixture) were conducted between 2–4 years after the start of the study in a facility with trained personnel with staged, monitored standard methods. Each food challenge consisted of several escalating doses of the food protein in flour or powder form concealed in an appropriate vehicle, such as applesauce or pudding, ingested by the participant every 15 min as tolerated. Typically challenges started with 2 mg and escalated upto a max of 8 g of total food protein as per our validated methods [26,27,28].

Figure 2 indicates the results of oral food challenges (OFCs). The percent of participants able to consume 8 g of protein was significantly higher in all mixed protein groups compared to the controls (q < 0.05). There were 44, 14, and 14 participants who had available OFC outcomes in the control, peanut, and mixture high groups, respectively. Interestingly, results from questionnaires demonstrated that those on the mixture diet were more apt to diversify the diet of their infant compared to single, double, and control groups (Appendix A Table A1).

**Figure 3:**
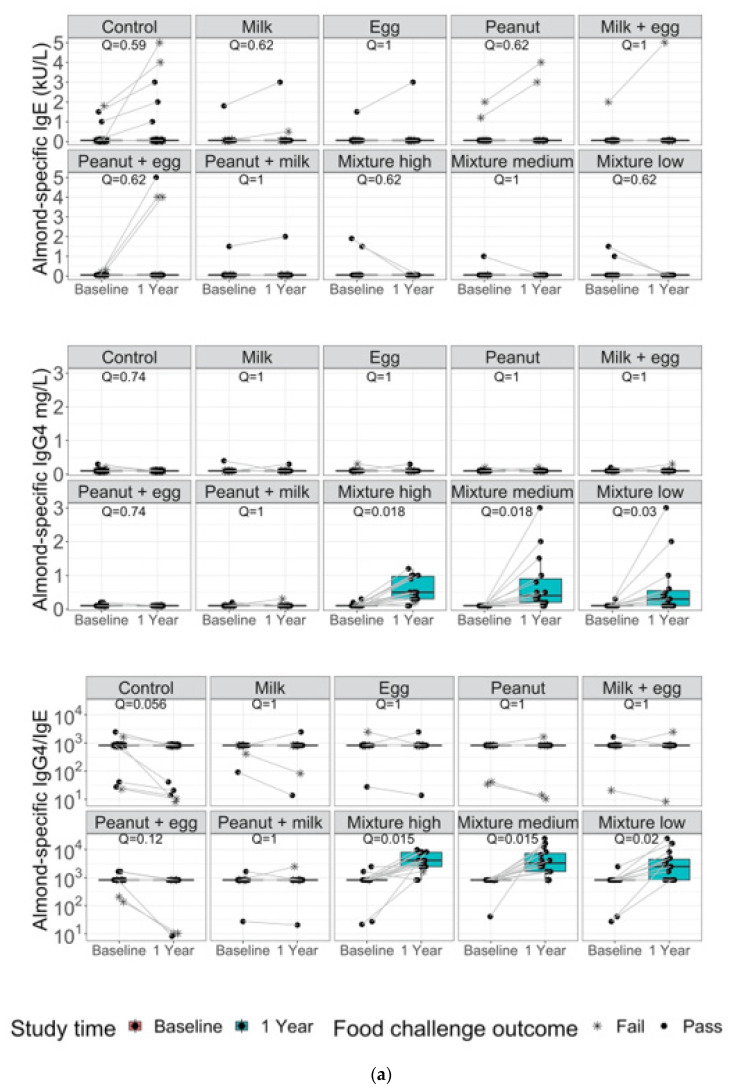

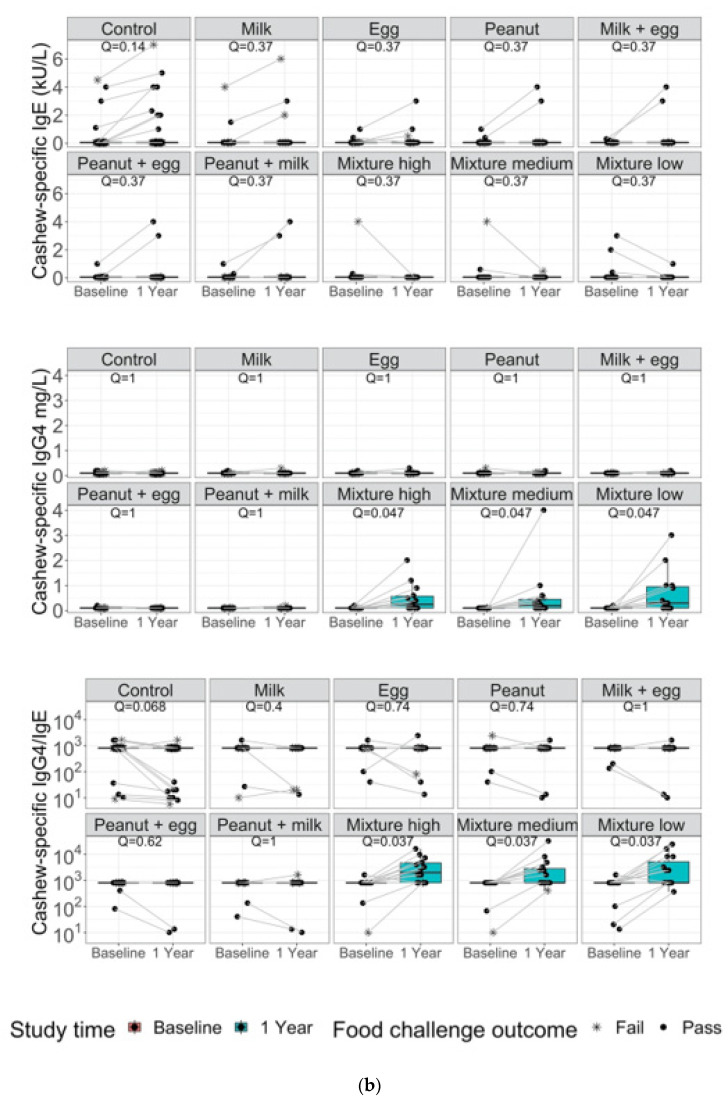

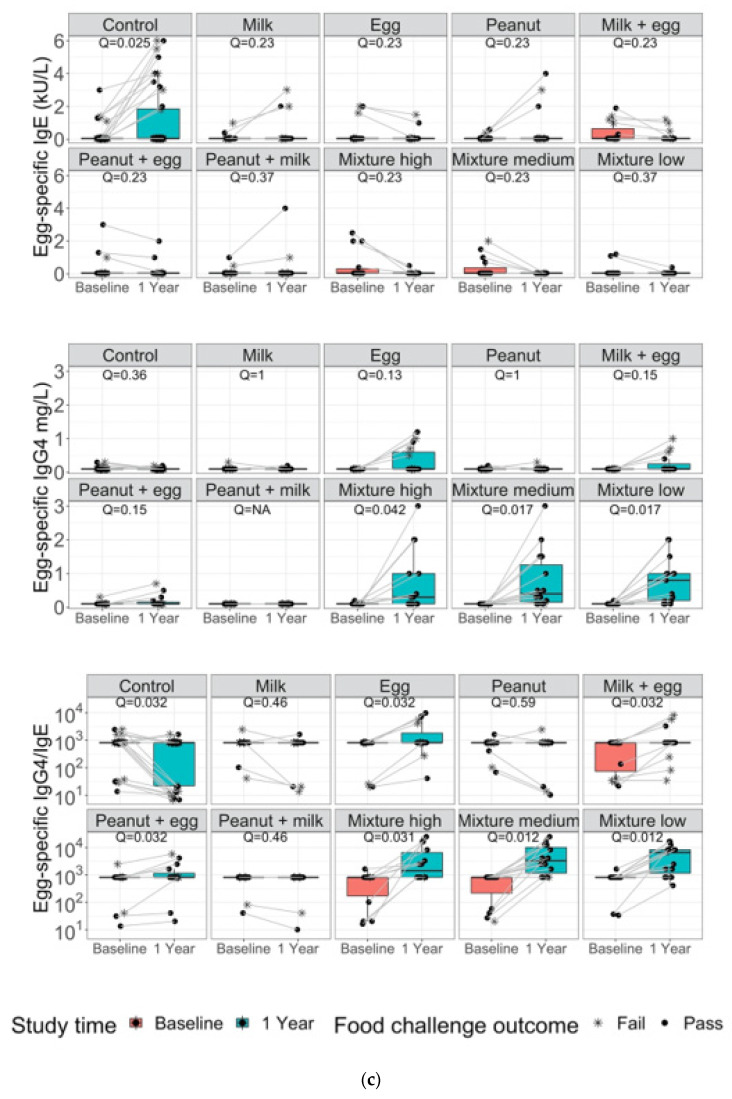

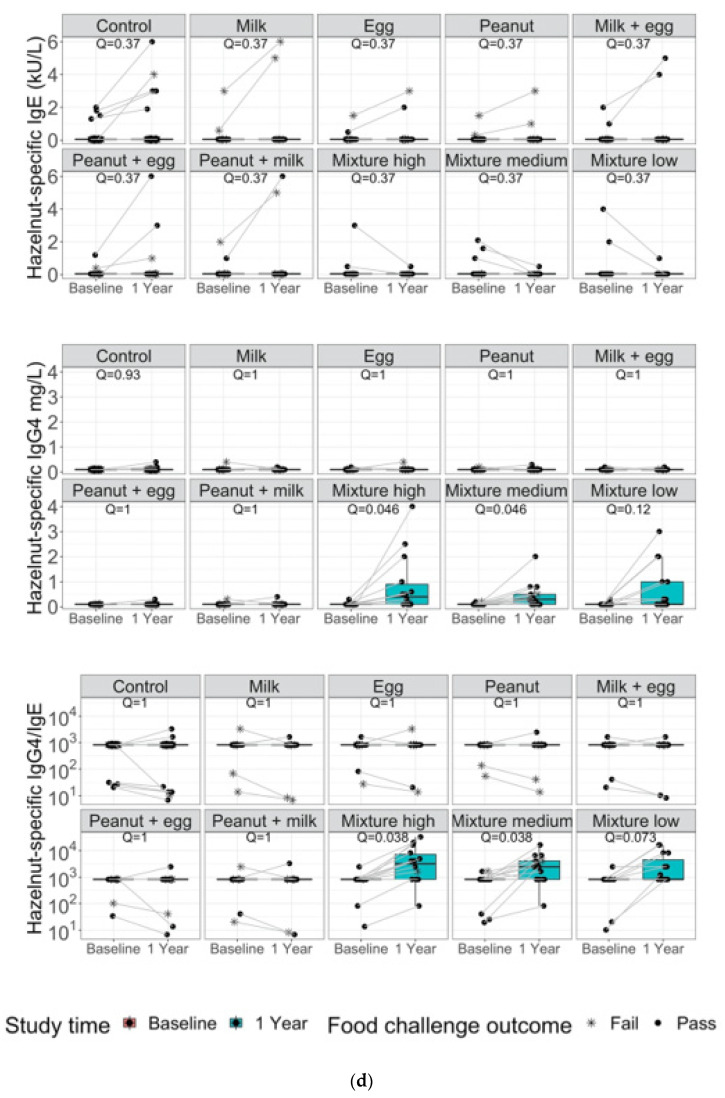

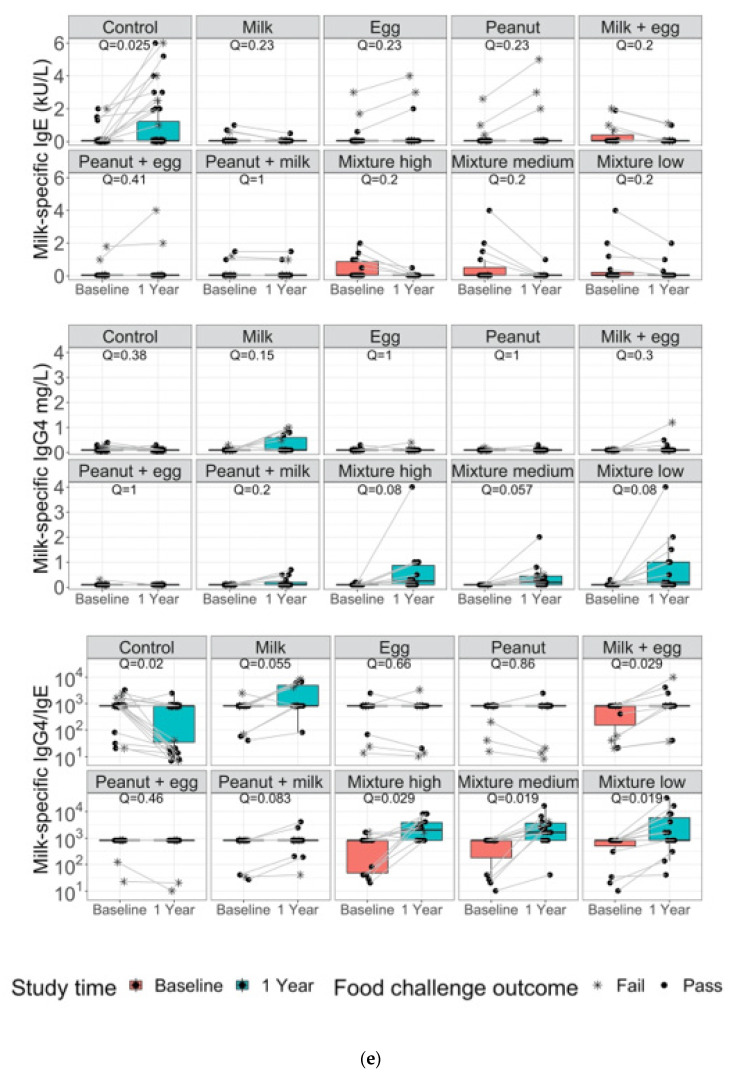

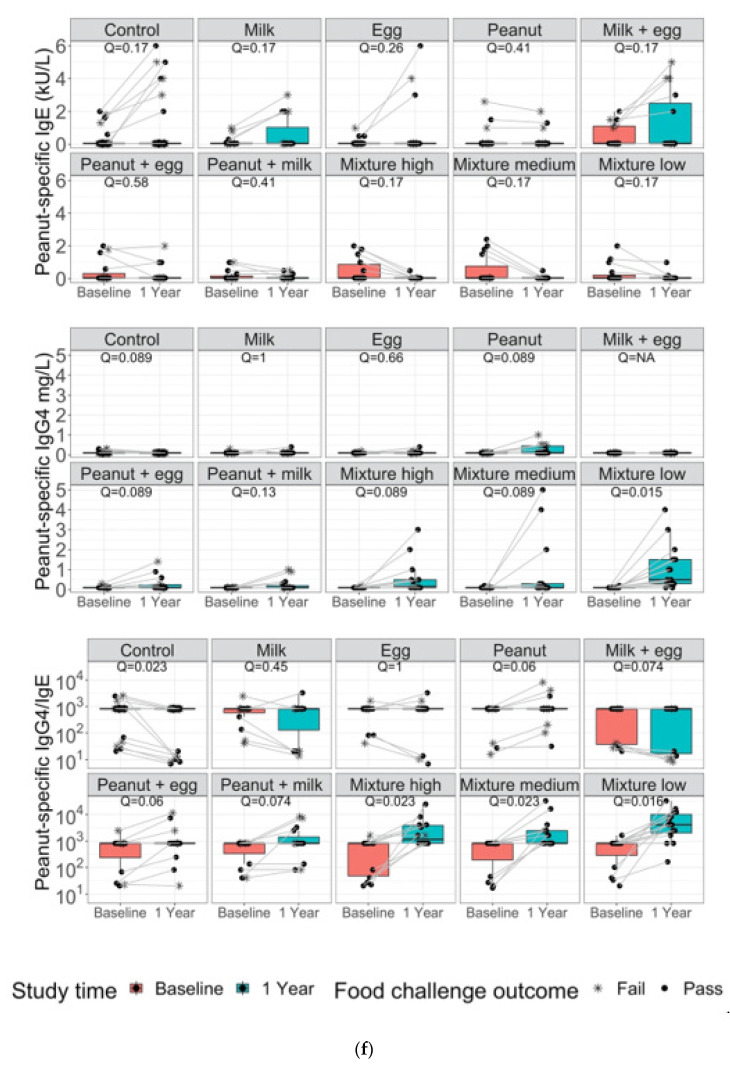

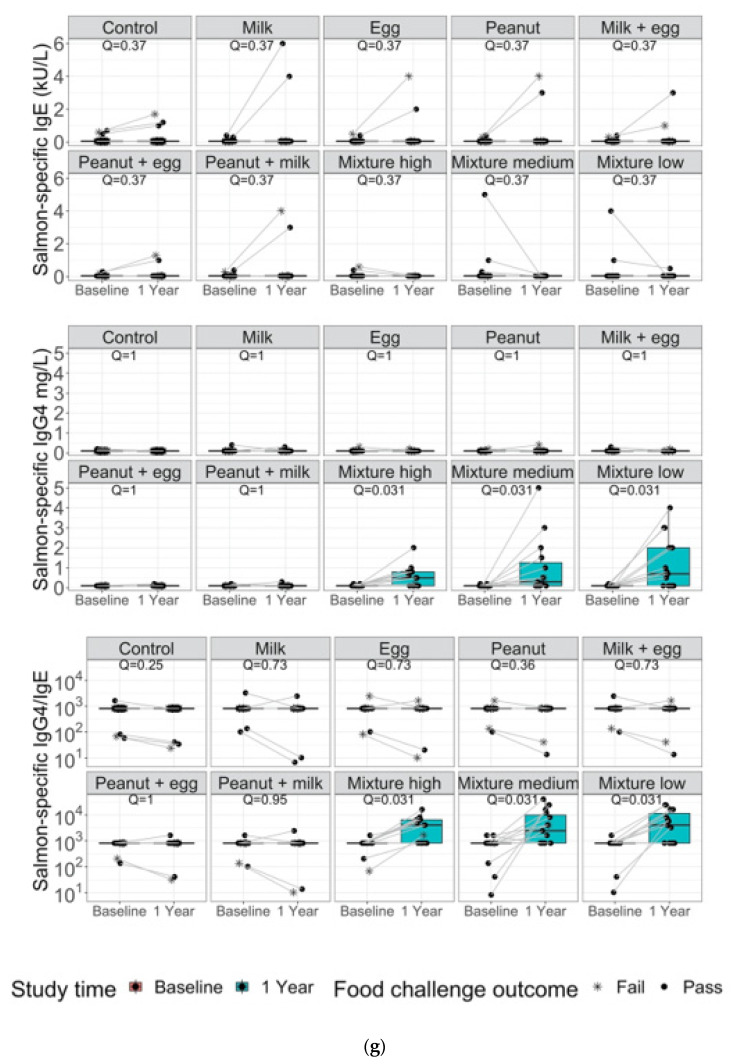

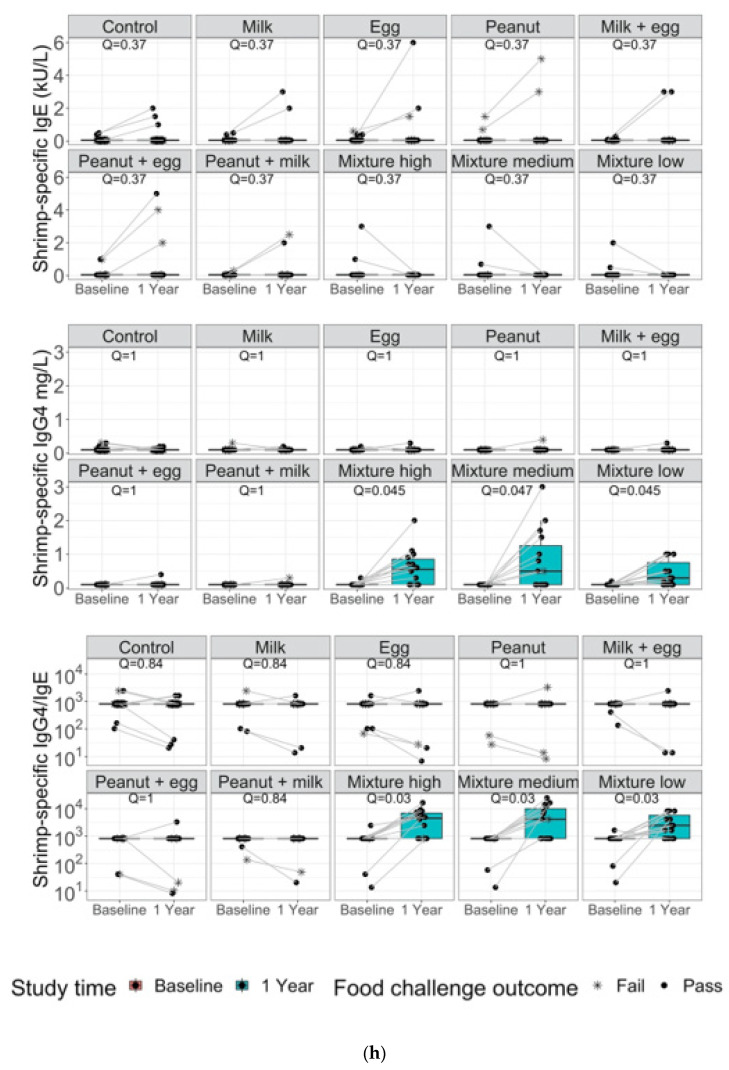

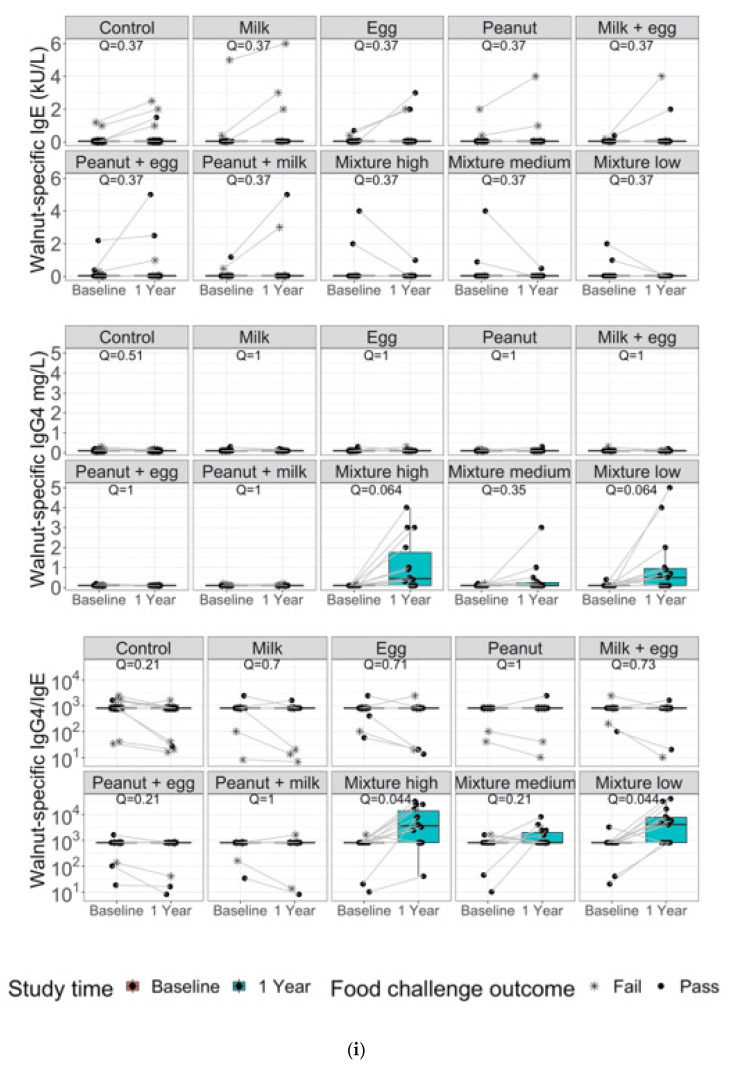

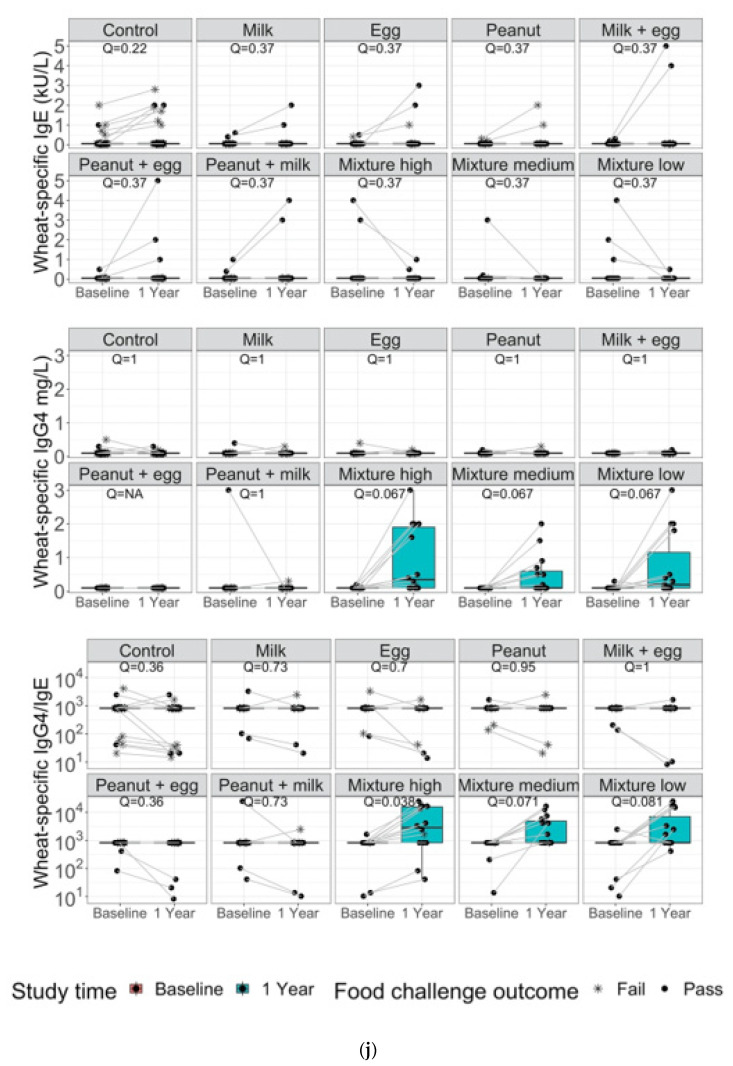
Allergen-specific IgG4, IgE and IgG4/IgE ratios for (**a**) Almond; (**b**) Cashew; (**c**) Egg; (**d**) Hazelnut; (**e**) Milk; (**f**) Peanut; (**g**) Salmon; (**h**) Shrimp; (**i**) Walnut and (**j**) Wheat are compared for controls, single, double, and multi-allergen groups (high, medium, and low) at baseline and after one year from the start of the study.

Specific IgG4, sIgE and IgG4/IgE ratios for all 10 allergens are shown in Figure 3a–j. The IgG4/IgE ratios for all mixed protein groups (low, medium, and high) were significantly higher at end of study compared to baseline (q < 0.1), even for peanut, egg, or milk compared to single early intervention active arms. The increase in the ratio can be mainly attributed to increases in IgG4 concentration rather than decreases in IgE. Peanut, milk, and egg biomarkers show improvement compared to control, however specificity was demonstrated only with peanut, milk, and egg containing food, respectively.

## 4. Discussion and Conclusions

While a number of studies have established the safety and efficacy of the early introduction of single allergens in infants for the prevention of FA, few studies have evaluated the dose, timing, and number of allergens that can be safely introduced in infants, particularly in those with eczema or those at high risk of atopy [29]. We therefore conducted a study to determine the safety and efficacy of the early introduction of single, double, and multiple allergens in infants for the prevention of FAs.

Our data from this pilot study suggest that consumption of single, double, and multiple allergens daily in infants is likely safe at up to 3000 mgs of protein. No increases in the incidence of eczema were observed in infants in the control or active groups, even in infants who had eczema at enrollment or those at high risk of atopy. The safety of multiple allergen introduction has been supported by a few other studies. The Holl study fed infants up to 16 allergens simultaneously with no adverse events. However, it should be noted that in the Holl study, the total protein amount in the allergen mix was only 480 mg, an amount much lower than the highest amount used in our study. Furthermore, the study only recruited healthy infants with no severe eczema. The EAT study, which also concluded that the early introduction of multiple allergens in infants was safe, differed from our study in that multiple allergens were introduced sequentially rather than simultaneously, and infants were recruited from the general population. The amount used in the EAT study (2000 mgs) was also lower than that used in our study (3000 mgs). The study by Cox et al. found infrequent reactions (0.1%) to early introduction of allergenic products similar or lower to what would be expected in a typical US infant population, indicating that early introduction with low doses is within safety parameters for typical feeding [30].

In our study, each food challenge consisted of several escalating doses of the food protein in flour or powder form concealed in an appropriate vehicle, such as applesauce or pudding, ingested by the participant every 15 min as tolerated. Typically, challenges started with 2 mg and escalated upto a max of 8 g of total food protein as per our validated methods [26,27,28]. Results from these food challenges as well as biomarker data from our study suggest that a daily dose of multiple allergens may be efficacious for the prevention of FA, even at low amounts (total 300 mg or 30 mg of each protein). This amount was found to be sufficient for decreasing positive food challenge responses and increasing specific food allergens IgG4/IgE. Our study found no difference in sIgE levels for all ten allergens between baseline and one year in the active groups. However, the sIgG4/sIgE ratio for all 10 allergens were significantly increased for mixtures (low, medium, and high) at the one-year time point compared to baseline. The change in the sIgG4/sIgE ratio was due to increases in IgG4 rather than decreases in sIgE. The results of this study are similar to other studies which have shown that sIgE and sIgG4 are indicative of allergy and tolerance, respectively. In the LEAP study, peanut-specific IgG4 increased in the peanut consumption group while a greater percentage of participants in the avoidance group had elevated titers of peanut-specific IgE [11]. A large randomized trial of egg introduction from 4 months of age in infants at risk for egg allergy found that levels of IgG4 to egg proteins and IgG4/IgE ratios were higher in those randomized to egg than in controls at 12 months; however, there was no significant difference in egg-specific sIgE levels at 12 months [31]. Numerous immunotherapy studies in children for FA have also found an increased ratio of sIgG4/sIgE to be indicative of tolerance [26,32,33,34,35,36]. Overall, the results from this pilot study demonstrate superior efficacy in food challenge outcomes in those individuals fed with the multiple mixture vs. single and vs. double as early introduction. A possible reason why the efficacy data shown in our study for multiple allergens is improved over single or double food early intervention could be due to a synchronous effect in inducing tolerance. It has been hypothesized that consumption of multiple allergens simultaneously increases polyclonal T cell memory subsets, increasing Th1 receptor diversity, leading to enhanced protection against potentially allergenic proteins compared to double or single food early intervention [37].

We believe regular daily dosing of small amounts of mixed food proteins was key to the success of this study. Participants reported that remembering to eat the powder mixture daily in small amounts was easier than if they were to dose 2–3 times a week with a larger serving of many foods. In comparison, in the EAT study [18], the mixture of allergens was prepared at patients’ homes; individually prepared allergen mixes could be associated with dosing/serving size variations. Use of premixed allergens as used in this study would reduce this variability. Lastly, families who had taken the mixture of foods as an early intervention felt more comfortable having their children eat table foods. 

The results of this pilot study suggest that the consumption of small convenient amounts of multiple allergens daily (even those with eczema or those at high risk of atopy) for the prevention of food allergies may be safe and efficacious. Larger, randomized controlled studies are needed to confirm these results. Although 3000 mg of allergenic protein was found to be safe, this high amount is impractical and can lead to noncompliance [19]. It would not impede optimal nutrient intake in the infant per USDA feeding guidelines for infants. The low mixture amount (300 mg protein) used in our study does not add significant calories and is not a substitute for foods, but instead the clinical data here shows that it can be easily used with breast feeding and/or table foods when the child is ready.

## 5. Patents

The following patent is associated with this work: “Special Oral Formula for Decreasing Food Allergy Risk and Treatment for Food Allergy”.

## Figures and Tables

**Table 1:** Summary of Analyses with Reference Methodology.

Category	Analysis	Reference Method
Protein Characterization	SDS-PAGE	Bio-Rad Laboratories or Thermofisher Scientific—Pierce Protein Methodology
Protein Quantitation	Kjeldahl Titration	AOAC 991.20
Bioburden	Total Aerobic Microbial Count—Pour Plate	USP/NF <61> or FDA BAM
	Total Yeast and Mold Count—Pour Plate	USP/NF <61> or FDA BAM
	*Escherichia coli*—Direct Inoculation	USP/NF <62> or FDA BAM
	*Salmonella species*—Direct Inoculation	USP/NF <62> or FDA BAM
	*Listeria species*—Absent/Present	AOAC-RI 061702
	*Listeria monocytogenes*—Absent/Present	AOAC-RI 061701
	*Listeria species* Confirmation (Genus 48 h)	FDA BAM Chapter 10
	Aflatoxin Panel	AOAC 2005.8 or Modified AOAC 999.07

SDS-PAGE—Sodium dodecyl sulfate polyacrylamide gel electrophoresis; USP/NF—United States Pharmacopeia/National Formulary; AOAC-RI—Association of Official Analytical Chemists—Research Institute; FDA BAM—Food and Drug Administration Bacteriological Analytical Manual.

**Table 2:** Summary of Total Protein and Marker Protein Acceptance Criteria.

Allergen	Marker Protein	Published Weight (kDa)	Observed Reference Data		Initial Acceptance Criteria, Inclusive		
			Weight (kDa)	Intensity (10 µg Load)	Weight (kDa)	Intensity (10 µg Load)	Total Protein
Almond	Pru du 6 (Amandin)	40	38.5–41.7	1,142,948–4,526,868	34.7–45.9	800,064–5,884,928	10–70%
Cashew	Ana o 2 (Anacardein)	33	26.5–34.1	752,512–6,028,437	23.9–37.5	526,758–7,836,968	10–55%
Egg	Gal d 2 (Ovalbumin)	43–45	39.0–44.6	6,627,824–14,047,568	35.1–49.1	4,639,477–18,261,838	50–95%
Hazelnut	Cor a 9 (Corylin)	35–40	31.7–34.8	1,963,532–6,958,956	28.5–38.3	1,374,472–9,046,643	10–65%
Milk	Bos d 5 (β-Lactoglobulin)	18–18.3	14.7–16.9	1,007,988–4,842,720	13.2–18.6	705,592–6,295,536	10–65%
Peanut	Ara h 3 (Glycinin)	37	35.0–40.4	1,125,819–3,025,396	31.5–44.4	788,073–3,933,015	15–75%
Salmon	Sal s 2 (β-enolase)	47.3	41.2–47.4	147,088–529,396	37.1–52.1	102,962–688,215	35–99%
Shrimp	Pen a 1 (Tropomyosin)	36	38.7–38.8	953,381–1,453,936	34.8–42.7	667,367–1,890,117	60–99%
Walnut	Jug r 4 (11S globulin)	30–40	29.6–36.2	1,211,022–6,709,672	26.6–39.8	847,715–8,722,574	10–75%
Wheat	Tri a 26 (Glutenin)	88	75.9–94.6	209,988–1,396,822	68.3–104.1	146,992–1,815,869	10–95%

**Table 3:** Patient demographics.

Demographic Characteristics at Baseline	Control *N* = 45	Active Milk (*N* = 15)	Active Egg (*N* = 15)	Active Peanut (*N* = 15)	Active Peanut/Milk (*N* = 15)	Active Milk/ Egg (*N* = 15)	Active Peanut/ Egg (*N* = 15)	Active Mixture Low (*N* = 15)	Active Mixture Medium (*N* = 15)	Active Mixture High (*N* = 15)
# Female	23	8	7	8	7	8	7	8	8	7
Age months (median and range)	6 (2–12)	5 (2–12)	5 (2–12)	6 (2–12)	5 (2–12)	5 (2–12)	6 (2–12)	6 (2–12)	6 (2–12)	5 (2–12)
Weight in kg (median and range)	7 (5–9)	7 (5–9)	6 (5–8)	7 (5–9)	6 (4–8)	6 (5–9)	7 (6–10)	7 (5–9)	8 (6–10)	7 (5–9)
# breast feeding until 6 mo	45	15	15	15	15	15	15	15	15	15
# no eczema (scored 0–9.9)	12	4	3	4	4	3	4	4	3	4
# Mild eczema (scored 10 to 28.9)	10	4	4	3	4	4	4	3	4	4
# Moderate eczema (scored 29.0 to 48.9)	15	4	4	4	4	4	4	4	4	3
# Severe eczema (scored 49.0 to 103)	8	3	4	4	3	4	3	4	4	4
# High risk * Family hx	23	7	8	8	7	8	7	8	7	8
Ethnicity										
Hispanic	4	2	2	1	1	1	2	1	1	1
Afro American	5	1	1	2	2	1	0	2	1	1
Caucasian	23	7	8	6	7	6	7	7	8	7
Pacific Islander	6	4	3	3	4	4	3	4	4	3
Asian	7	1	1	2	1	3	3	1	1	3

* High risk was defined as one first degree relative with FA/atopic dermatitis or two first degree relatives with atopic disease.

**Table 4:** Safety: Adverse reactions during study.

Characteristics	Control *N* = 45 at Baseline	Control Arm Reactions During Study	Active *N* = 135 at Baseline	Active Arm Reactions During Study
No eczema	8 (18%)	4 (all mild rash) (9%)	22 (16%)	11 (all mild rash) (8%)
Mild eczema	22 (49%)	1 (2%)	61 (45%)	2 (1%)
Moderate eczema	10 (22%)	none	35 (26%)	4 (3%)
Severe eczema	5 (11%)	1 (2%)	17 (13%)	none
High risk * Family hx	*N* = 23 (51%)	1 (2%)	*N* = 69 (51%)	3 (2%)
Other adverse events within 2 h of serving		Control arm reactions during study		Active arm reactions during study
Vomiting		0		0
Diarrhea		0		0
Anaphylaxis (more than two organ systems involved)		0		0
Wheezing		0		0
Cough		0		0
Epinephrine use		0		0

* High risk was defined as one first degree relative with FA/atopic dermatitis or two first degree relatives with atopic disease.

**Table 5:** Percentage of participants consuming real foods at beginning and end of study.

Food Item Consumed by Participant at Start of Study	Participants Consuming Food Item at Start of and during the Study		Participants Consuming All 10 Food Items as Table Foods at End of 1st Year	
	*N*	%	*N*	%
Milk	15	100	6	40
Egg	15	100	5	33
Peanut	15	100	6	40
Milk/egg	15	100	7	47
Egg/peanut	15	100	7	47
Milk/peanut	15	100	8	53
Milk/egg/peanut/cashew/almond/shrimp/walnut/wheat/fish/hazelnut (300 mg)	15	100	15	100
Milk/egg/peanut/cashew/almond/shrimp/walnut/wheat/fish/hazelnut (900 mg)	15	100	15	100
Milk/egg/peanut/cashew/almond/shrimp/walnut/wheat/fish/hazelnut (3000 mg)	15	100	15	100

## Data Availability

The data presented in this study are available on request from the corresponding author. The data are not publicly available as the patent is owned by a company and Stanford and permission is needed before release of data.

## Appendix A

**Table A1:** Number of infants fed more than 10 foods as table foods by one year of age.

Allergen Group	*N*
Mixture (low dose)	15 (100%)
Mixture (medium dose)	14 (93%)
Mixture (high dose)	15 (100%)
Control	7 (15.5%)
Peanut	10 (66.7%)
Milk	8 (53.3%)
Egg	9 (60.0%)
Peanut/milk	11 (73.3%)
Milk/egg	10 (66.7%)
Egg/peanut	8 (53.3%)
