# Correction: Quake et al. Early Introduction of Multi-Allergen Mixture for Prevention of Food Allergy: Pilot Study. *Nutrients* 2022, *14*, 737

**DOI:** 10.3390/nu15010135

**Published:** 2022-12-28

**Authors:** Antonia Zoe Quake, Taryn Audrey Liu, Rachel D’Souza, Katherine G. Jackson, Margaret Woch, Afua Tetteh, Vanitha Sampath, Kari C. Nadeau, Sayantani Sindher, R. Sharon Chinthrajah, Shu Cao

**Affiliations:** Department of Medicine, Division of Pulmonary, Allergy and Critical Care Medicine, Sean N. Parker Center for Allergy and Asthma Research at Stanford University, Stanford, CA 94304, USA

## 1. Error in Figure 1

In the original publication [1], there was an error in **Figure 1**. **Change food challenge N = 15 for control to N = 45**. The corrected **Figure 1** appears below.

## 2. Error in Figure 2

In the original publication, there was an error in **Figure 2**. **Change the numbers of the sample sizes and Q value**. The corrected **Figure 2** appears below. 

## 3. Text Correction

There was an error in the original publication. **The percent of participants able to consume 8 g of protein was significantly higher in all mixed protein groups compared to controls (q < 0.01)**.

A correction has been made to **Results**, **page 8**:

**The percent of participants able to consume 8 g of protein was significantly higher in all mixed protein groups compared to the controls (q < 0.05). There were 44, 14, and 14 participants who had available OFC outcomes in the control, peanut, and mixture high groups, respectively**.

The authors apologize for any inconvenience caused and state that the scientific conclusions are unaffected. This correction was approved by the Academic Editor. The original publication has also been updated.

## Figures and Tables

**Figure 1:**
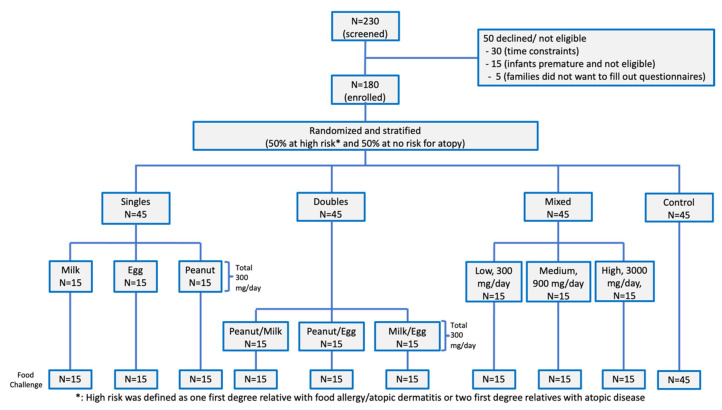
Consort diagram. 180 participants were randomized into three active and one control group. The active phase of the study was for one year and there were no dropouts. Single foods (milk, egg, or peanut); two foods (milk/egg, egg/peanut, milk/peanut), Mixed (milk/egg/peanut/cashew/almond/shrimp/walnut/wheat/salmon/hazelnut at low, medium, or high doses).

**Figure 2:**
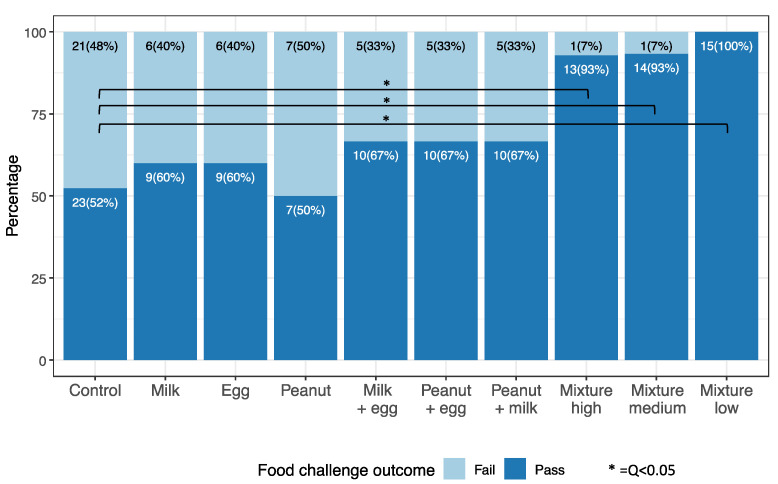
Oral Food Challenges: Food challenge outcome in active (singles, doubles, and mixtures) and control groups 2–4 years after start of study. Oral food challenges (up to 8 g of total protein from the 10-food allergen mixture) were conducted between 2–4 years after the start of the study in a facility with trained personnel with staged, monitored standard methods. Each food challenge consisted of several escalating doses of the food protein in flour or powder form concealed in an appropriate vehicle, such as applesauce or pudding, ingested by the participant every 15 min as tolerated. Typically challenges started with 2 mg and escalated upto a max of 8 g of total food protein as per our validated methods [26–28].
